# Pharmacological Inhibition of Endogenous Hydrogen Sulfide Attenuates Breast Cancer Progression

**DOI:** 10.3390/molecules27134049

**Published:** 2022-06-23

**Authors:** Nazeer Hussain Khan, Di Wang, Wenkang Wang, Muhammad Shahid, Saadullah Khattak, Ebenezeri Erasto Ngowi, Muhammad Sarfraz, Xin-Ying Ji, Chun-Yang Zhang, Dong-Dong Wu

**Affiliations:** 1 Henan International Joint Laboratory for Nuclear Protein Regulation, School of Basic Medical Sciences, Henan University, Kaifeng 475004, China; kakakhan3514@gmail.com (N.H.K.); wangdi20220316@gmail.com (D.W.); saadullah271@gmail.com (S.K.); ebenezerngowi92@gmail.com (E.E.N.); chiefpharm@gmail.com (M.S.); 2 School of Life Sciences, Henan University, Kaifeng 475004, China; 3 Department of Breast Surgery, The First Affiliated Hospital, Zhengzhou University, Zhengzhou 450052, China; victor1125@foxmail.com; 4 Department of Biological Sciences and Biotechnology, Faculty of Science and Technology, Universiti Kebangsaan Malaysia, Bangi 43600, Malaysia; mshahdaslam@gmail.com; 5 Department of Biological Sciences, Faculty of Science, Dar es Salaam University College of Education, Dar es Salaam 11101, Tanzania; 6 Faculty of Pharmacy, The University of Lahore, Lahore 54590, Pakistan; 7 Kaifeng Key Laboratory of Infection and Biological Safety, College of Medicine, Henan University, Kaifeng 475004, China; 8 Department of Thoracic Surgery, The First Affiliated Hospital of Zhengzhou University, Zhengzhou 450052, China; 9 Department of General Thoracic Surgery, Hami Central Hospital, Hami 839000, China; 10 School of Stomatology, Henan University, Kaifeng 475004, China

**Keywords:** endogenous hydrogen sulfide, breast cancer, apoptosis, signaling pathway, tumor growth

## Abstract

Hydrogen sulfide (H_2_S), a gaseous signaling molecule, is associated with the development of various malignancies via modulating various cellular signaling cascades. Published research has established the fact that inhibition of endogenous H_2_S production or exposure of H_2_S donors is an effective approach against cancer progression. However, the effect of pharmacological inhibition of endogenous H_2_S-producing enzymes (cystathionine-γ-lyase (CSE), cystathionine-β-synthase (CBS), and 3-mercaptopyruvate sulfurtransferase (3-MPST)) on the growth of breast cancer (BC) remains unknown. In the present study, DL-propargylglycine (PAG, inhibitor of CSE), aminooxyacetic acid (AOAA, inhibitor of CBS), and L-aspartic acid (L-Asp, inhibitor of 3-MPST) were used to determine the role of endogenous H_2_S in the growth of BC by in vitro and in vivo experiments. An in silico study was also performed to confirm the results. Corresponding to each enzyme in separate groups, we treated BC cells (MCF-7 and MDA-MB-231) with 10 mM of PAG, AOAA, and L-Asp for 24 h. Findings reveal that the combined dose (PAG + AOAA + L-Asp) group showed exclusive inhibitory effects on BC cells’ viability, proliferation, migration, and invasion compared to the control group. Further, treated cells exhibited increased apoptosis and a reduced level of phospho (p)-extracellular signal-regulated protein kinases such as p-AKT, p-PI3K, and p-mTOR. Moreover, the combined group exhibited potent inhibitory effects on the growth of BC xenograft tumors in nude mice, without obvious toxicity. The molecular docking results were consistent with the wet lab experiments and enhanced the reliability of the drugs. In conclusion, our results demonstrate that the inhibition of endogenous H_2_S production can significantly inhibit the growth of human breast cancer cells via the AKT/PI3K/mTOR pathway and suggest that endogenous H_2_S may act as a promising therapeutic target in human BC cells. Our study also empowers the rationale to design novel H_2_S-based anti-tumor drugs to cure BC.

## 1. Introduction

Breast cancer (BC) is a prevalent and growing concern of malignancy in women worldwide [1]. According to recent reports, approximately 2.3 million individuals are diagnosed with BC each year, with an annual mortality rate of around 450,000 [2,3]. Leading associated risk factors of BC include age, the aggravate prevalence of genetic mutations in predisposition genes (*BRCA1* and *BRCA2*), lifestyle base-modified (non-genetic) risk factors, nulliparity, early menarche, first pregnancy in women older than 30 years of age, older age onset of menopause, usage of oral contraceptives, and personal or familial history of BC and other clinical complaints [4,5,6].

Hydrogen sulfide (H_2_S), together with nitric oxide (NO) and carbon monoxide (CO), is involved in modulating multiple physiological and pathological processes. It has been widely regarded as an endogenous gasotransmitter molecule [7]. Under normal physiological conditions, three enzymes that are widely expressed in mammalian tissues and cells—namely cystathionine-β-synthase (CBS), cystathionine-γ-lyase (CSE), and 3-mercaptopyruvate sulfurtransferase (3-MST)—produce H_2_S [8]. H_2_S exhibits a pleiotropic and often dose-dependent effect after being released in the form of acid-labile sulfur and bound sulfane sulfur [9]. It has been well established that H_2_S is involved in mediating essential cellular mechanisms and plays a crucial role in the regulation of many physiological conditions, including energy production, neuroprotection, vasorelaxation, glucose homeostasis, and angiogenesis [10,11,12]. In particular to the role of H_2_S in cancer, it has been reported that endogenous H_2_S is involved in cancer development and progression [13,14].

In terms of cytoprotective biological response, H_2_S is considered to be a bidirectional target in cancer research. On the one hand, studies have shown that exogenous exposure of H_2_S donors prevents tumor development [15,16]. On the other hand, a number of studies have shown that reducing H_2_S levels by downregulating the production of endogenous H_2_S in cancer cells leads to a decline in cancer progression [17,18]. With these potent therapeutic characteristics in cancer research, H_2_S has gained the attention of many researchers, and drugs that can repress or trigger the synthesis or promote the release of H_2_S have attained enormous value in preclinical and clinical settings.

To facilitate the future clinical translation research of H_2_S and the proposition of promising anticancer approaches for therapeutic manipulation of H_2_S, a variety of novel inhibitors have been synthesized to suppress the activity of H_2_S-producing enzymes. Currently, three pharmacologically well-characterized compounds—namely DL-propargylglycine (PAG), L-aspartic acid (L-Asp), and aminooxyacetic acid (AOAA)—are considered to be competitive, potent inhibitors of CSE, 3-MPST, and CBS, respectively. PAG (C_5_H_7_NO_2_) is a selective inhibitor compound which basically blocks the active site of the CSE enzyme, thereby preventing it from binding to its original substrate. The solubility of this drug is significantly high in both water and PBS [19,20]. L-Aspartic acid (L-Asp) (C4H_14_NO_4_) works in similar manner to PAG. The solubility of the drug is relatively low—nearly half that of PAG in water [21]. Similarly, in many studies, AOAA has also been reported to reduce H_2_S synthesis by inhibiting the function of CBS in both acidic and in prodrug forms. AOAA has been reported to reduce intracellular adenosine triphosphate levels and decrease glycolysis rate, which in doing so, regulates cellular activities. Studies have claimed the selectivity of AOAA; however, it also inhibits CSE. The effect of AOAA is concentration dependent. In addition, AOAA is highly soluble in both water and PBS [20,22,23]. Given the encouraging results in inhibiting H_2_S synthesis, it is worth investigating the electrostatics (i.e., protonation or deprotonation states) of these drugs both in solution and physiological condition.

These inhibitors have been well studied in various experimental and clinical studies as potential future anti-cancer therapies [23,24,25].

However, there is no study on the pharmacological inhibition of H_2_S in BC cells using these inhibitors. Therefore, by using PAG, L-Asp, and AOAA as potent inhibitors of H_2_S-producing enzymes, the present research focused on investigating the effect of pharmacological inhibition of endogenous H_2_S on human BC cell proliferation, invasion, and migration by using various in vitro and in vivo approaches.

## 2. Material Method

### 2.1. Cell Culture

Human BC cell lines MCF-7 and MDA-MB-231 were purchased from Fenghbio Biosciences (Changsha, Hunan, China). The cells were grown in DMEM media supplemented with 10% fetal bovine serum, 100 ug/mL streptomycin, and 100 U/mL penicillin and were maintained at 37 °C in a humidified atmosphere of 5% CO_2_ and 95% air.

### 2.2. Drugs Formulations/Treatment

To obtain a concentration of drugs (DL-propargylglycine (PAG, inhibitor of CSE), aminooxyacetic acid (AOAA, inhibitor of CBS), and L-aspartic acid (L-Asp, inhibitor of 3-MST)) that inhibited cellular H_2_S synthesis but was noncytotoxic to cell survival, cells were treated with a gradient of concentrations (1, 2, 5, 10, and 20 mM) for 24 h. Followed by the 24 h treatment of individual drugs, optical density (OD) of the treated cells using a CCK-8 kit was measured. Drug concentrations and cell toxicity response were measured for all treatments. Additionally, the combined dose effects of three inhibitors were investigated at a concentration of 10 mM and a higher concentration of individual drugs (30 Mm) by using a CCK-8 test. Phosphate buffer saline (PBS) was used to treat the control group cells. 

### 2.3. Cell Viability Assay

The CCK-8 assay was used to detect cell viability according to the manufacturer’s instructions. In brief, 10,000 MCF-7 and MDA-MB-231 cells were seeded in 96-well plates and cultured in normal medium for 24 h after the treatment. At the check point, 10% CCK-8 solution was added to each well of the 96-well plate before OD readings, as done in previous studies [26].

### 2.4. Detection of H_2_S Level

To determine the inhibitory efficacy of drugs after the 24 h treatment, the enzyme-linked immunosorbent assay kit (LanpaiBio, Shanghai, China) was used to detect H_2_S levels in MCF-7 and MDA-MB-231 cells [27]. The cell culture supernatants were collected to test the levels of H_2_S. Next, the standard controls were prepared accordingly, and samples were treated with reagents and incubated for 0.5 h at 37 °C. Then, color-developing agents were added to each well and incubated for 15 min at 37 °C. The OD of each well was measured with a microplate reader at 450 nm. Using the equation derived from the standard controls and the absorbance of the samples obtained from the microplate reader, the concentration of H_2_S was determined. The experiments were repeated three times.

### 2.5. Cell Proliferation Assay

For assessment of cell proliferation, the Light EdU Apollo 567 in Vitro Imaging Kit (RiboBio, Guangzhou, Guangdong, China) was used to determine the proliferation. In brief, 10^4^ cells/well were seeded in a 96-well plate, incubated, and treated with corresponding drugs for 24 h. After the treatment, the growth media was replaced with pure media containing EdU A and incubated for 2 h. Then, cells were fixed with 4% paraformaldehyde (PFA) and were washed with PBS. By adopting the kit protocol, the Apollo step was conducted under dark conditions. After washing with PBS and methanol, cells were treated with DNA staining (Hoechst reagent) under dark conditions at room temperature. Imaging was performed using a fluorescent microscope, and the proliferation rate was calculated using the following formula: Percentage cell proliferation = (EdU − positive cells/total cells × 100).

### 2.6. Wound Healing Assay

The scratch wound-healing motility assay was performed to evaluate the migration ability of treated BC cells. In a 6-well plate, after the cells reached 80–90% confluence, a sterile pipette tip was scraped across the monolayer cells. The cells were then returned to the incubator after the drug treatment until the indicated time. An Olympus CKK41 microscope was used to photograph the reprehensive sites, which were analyzed by ImageJ software to measure the migration. The migration rate (MR) was estimated using the following formula: MR (%) = [(A − B)/A] × 100, where A and B are the widths at 0 and 24 h, respectively.

### 2.7. Colony Formation Assay

To assess the effect of drug treatment on colony formation, we seeded the cell lines (5 × 10^2^) into 6-well plates and cultured them for 14 days at 37 °C. At the end of experiment, methanol was used to fix the colonies, which were then stained at room temperature with 0.5% crystal violet. The colony number was then calculated by scanning the plate.

### 2.8. Migration and Invasion Assay

To determine the migration and invasion ability of treated cells, we firstly spread the media and cell suspension (200 μL) in the upper chamber of a 24-well specialized plate. The lower chamber contained 20% fetal bovine serum (600 μL), used as a chemoattractant. After incubation for 24 h, cells were fixed with methanol for 20 min and then stained with crystal violet dye solution for 40 min. Finally, images for transwell invasion were taken using a Zeiss Axioskop 2 plus microscope (Carl Zeiss, Thornwood, NY, USA).

### 2.9. Cell Death Assay 

For the detection of apoptosis/necrosis of 24 h treated BC cells, TDT-mediated dUTP-biotin nick end labeling (TUNEL) assay was performed. In brief, 1 × 10^5^ cells/well seeded in a 96-well plate were treated and incubated for 24 h. Cells were washed with PBS once and fixed with 4% PFA for 30 min at room temperature. After fixation, the cells were washed with PBS and treated with 1% triton for 5 min. Then, Tunel solution 50 μL was added per well and incubated for 80 min at 37 °C. After careful washing, cells were subjected to DAPI for 5 min and photographs were taken with a fluorescence microscope. Using ImageJ software, cell death rate was counted by using the ratio of TUNEL-positive cells in total cells.

### 2.10. Western Blot Analysis

The protein level was determined using a western blot. The principal antibodies (anti-B-cell lymphoma-extra-large (Bcl-xl), anti-B-cell lymphoma-2 (Bcl-2), anti-Bcl-xl/Bcl-2-associated death promoter (Bad), anti-Bcl-2-associated X protein (Bax), anti-cleaved poly adenosine diphosphate-ribose polymerase (PARP), anti-cleaved caspase-3, N and E cadherin, and Vimentin antibodies) were procured from ProteinTech (Chicago, IL, USA). Antibodies against PI3K/AKT/mTOR and their corresponding anti-phospho were obtained from cell Signaling technology (CST, Danvers, MA, USA), as the secondary antibody conjugated with horse. The internal control was chosen to be β-actin. An enhanced chemilumescence setup (Thermo Fisher Scientific, Rockford, IL, USA), was used to acquire the photographs. ImageJ software was used to calculate the band intensities.

### 2.11. Animal Study

The Henan University School of Medicine’s Committee on Medical Ethics and Welfare for the experimental Animals (HUSOM-2019-168) gave its approval for the animal research. With slight adjustments, the animal research was carried out as described earlier [28]. Vital River Laboratory Animal Technology Co., Ltd. (Beijing, China) provided the BALB/C nude mice (male, 4 weeks old). Nude mice’s right flanks were injected subcutaneously with MCF-7 and MDA-MA231 cells (5 × 10^6^ cells in 200 μL PBS). The mice were divided into five groups at random (*n* = 6 each group). For 28 days, 10 mM PAG, L-Asp, AOAA, and PAG + AOAA + L-Asp (combined group) were injected subcutaneously near the tumor. PBS was injected subcutaneously into the control animals. Every day, the body weights and tumor volumes were measured. Tumor volumes were calculated using the following formula: volume = length × width^2^/2 [29]. At the end of the trial, the mice were anaesthetized with 3% isoflurane and executed by cervical dislocation. The tumor growth inhibition rate (IR) was calculated as IR (%) = [(A − B)/A] × 100, where A and B represent the control and treatment groups’ respective average tumor weights [16].

### 2.12. Tumor Tissue Staining

Tumor specimens were fixed in paraffin embedded in 10% neutral-buffered formalin, and then tissues were segmented to thicknesses of 5 mm by adopting the hematoxylin and esosin (HE) staining procedures [26]. Images were taken with a Zeiss Axioskop 2plus microscope.

### 2.13. Immunohistochemistry (IHC)

The tumor microvessel density (MVD) was determined by using the Cluster of Differentiation 31 (CD31), a significant biomarker for vascular endothelial cells [30]. Tissues were stained with the CD31 antibody (CST), and tumor vessels were detected and measured with a Zeiss Axiokop 2plus microscope. In addition, tumor tissues were stained with anti-Ki67 antibody (CST), and Ki67 antibodies and positive cells were imaged using a Zeiss Axioskop 2 plus microscope. The proliferation index (PI) was calculated by dividing the number of Ki67-positive cells by the total number of cells [31]. Similarly, to find the apoptotic index (PI), tumor tissues were stained with the cleaved caspase-3 antibody. Cell death rate was calculated by the ratio of cleaved caspse-3-positive cells to the total cells [32].

### 2.14. Statistics Analysis

The mean ± standard error of the mean (SEM) was used to express all experimental data. The two-tailed Student’s t test was used to determine the difference between the two groups. Using SPSS 17.0 software, the difference between different groups was examined using one-way analysis of variance, followed by Tukey’s test. Statistical significance was defined as *p* < 0.05.

## 3. In silico Validation of Drugs/Inhibitors

### 3.1. Ligand and Receptor Protein Preparation

The structures of L-aspartic acid (L-Asp), DL-Propargylglycine (PAG), and aminooxyacetic acid (AOAA) in the Spatial Data File (SDF) were retrieved from the PubChem database (https://pubchem.ncbi.nlm.nih.gov/ (accessed on 20 December 2021)) with PubChem IDs 5960, 95575, and 286, respectively. Prior to PDBQT file generation, all the SDF files were prepared with the addition of Gasteiger charges and polar hydrogens by employing a set of AutoDock tools (version 1.5.6) and were saved in PDBQT format using the Open Babble tool integrated into PyRx software (version 0.8). The X-ray crystal structures of the target receptor proteins were retrieved from the Protein Data Bank (PDB) (https://www.rcsb.org/ (accessed on 21 December 2021)) with PDB IDs 3COG, 3OLH, and 4COO for Cystathionine gamma-lyase (CSE), 3-mercaptopyruvate sulfurtransferase (3-MPST), and Cystathionine beta-synthase (CBS), respectively. Before molecular docking, all the protein PDBs were preprocessed by removing the non-amino acid residues and water molecules, adding polar hydrogen atoms, and optimising by adding Kollman charges, utilizing the AutoDock tool [33,34].

### 3.2. Druggable Pockets and Molecular Docking Analysis

The druggable pockets of each selected protein receptor were identified through the P2RANK server (https://prankweb.cz/ (accessed on 26 December 2021)), which uses a template-independent machine learning-based method. The top-ranked predicted pockets were chosen and considered for the molecular docking simulation study. The detailed results of P2RANK are tabulated in Supplementary Table S1. The AutoDock Vina tool was utilized to execute molecular docking simulations due to its rapid, stochastic optimizations by operating multiple CPU cores, which allowed it to overcome the confines of the maximum number of rotatable bonds, atoms, and grid map size [35,36,37]. Docking grids of all macromolecule receptors were adjusted into squares of 24 Å with *x, y,* and *z* coordinates of −21.7641, 33.6885, and −18.7002 for Cystathionine gamma-lyase; −35.004, −20.3346, and −41.463 for 3-mercaptopyruvate sulfurtransferase; and −9.2621, 22.9917, and −18.5675 for Cystathionine beta-synthase, respectively, to define the ligand binding sites. The interaction of the ligand–protein complexes was visualized using the BIOVIA Discovery Studio Visualizer version 20.1.0 tool.

## 4. Results

### 4.1. Inhibition of Endogenous H_2_S Attenuates the Viability and Proliferation of Human BC Cells

**Figure 1:**
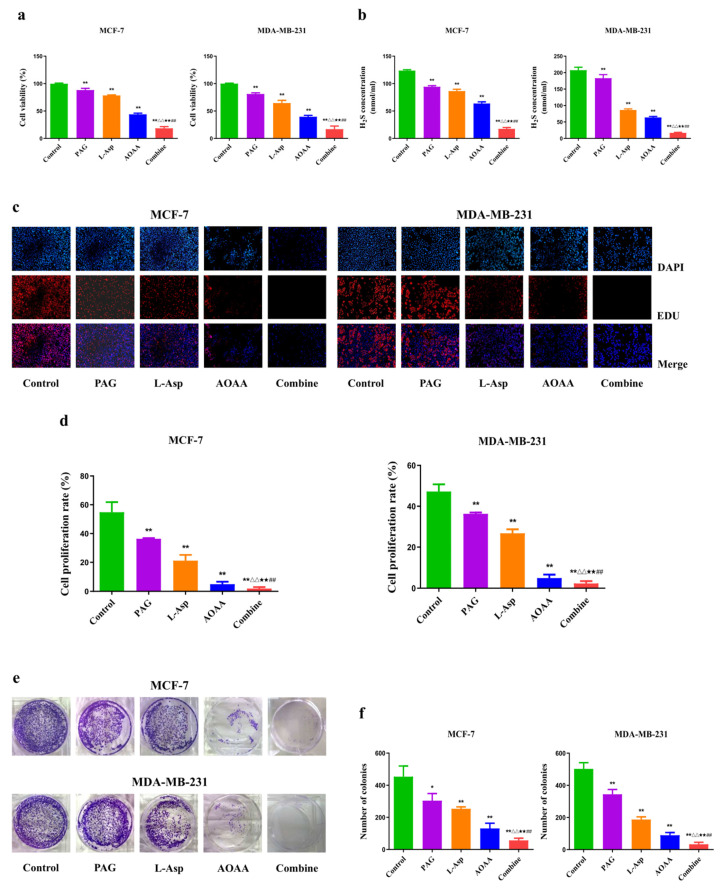
Effects of PAG, AOAA, and L-Asp on viability, H_2_S content, proliferation, and colony formation in human BC cells. (**a**) The CC-K8 assay was used to determine the percentage of viable cells. The cell viability of each group without PAG, AOAA, or L-Asp treatment was normalized as 100% and considered to be the control group. (**b**) The levels of H_2_S in BC cells were detected. (**c**) The proliferation rate of each group was analyzed. (**d**) DNA replication activities of BC cells in each group were examined by EdU assay (original magnification ×200). (**e**) The clonogenic capacity was determined in BC cells. (**f**) The number of colonies was calculated. The experiments were performed in triplicate. Data are presented as mean ± SEM. * *p* < 0.05, ** *p* < 0.01 compared with the control group; ^△△^ *p* < 0.01 compared with PAG group; ^★★^ *p* < 0.01 compared with AOAA group; ^##^ *p* < 0.01 compared with L-Asp group.

As presented in Figure 1, treatment of 10 mM of PAG, AOAA, and L-Asp reduced the cell viability in a dose-dependent manner, with a sharp decrease in H_2_S content in corresponding treated human cell (MCF-7 and MDA-MB 231) groups (Figure 1a,b). It was shown that there is a concentration-dependent decrease in the cell viability of cells (Supplementary Figure S1). Furthermore, the combined dose treatment (10 mM L-Asp + 10 mM PAG + 10 mM AOAA) had a stronger effect compared to treatment with 30 mM PAG and 30 mM L-Asp (Supplementary Figure S2). These preliminary findings on the drugs and cell viability suggest that the 10 mM treatment is the optimum dose for measuring the pharmacological effect on cells. Furthermore, this decrease in H_2_S content in treated cells led to a significant decline in the cell proliferation and colony formation ability of BC cells (Figure 1c–f). According to these results, the combined dose treatment and AOAA exhibit a strong inhibitory effect compared to L-Asp and PAG. Taken all together, these findings suggest that upon treatment with these drugs, either separately or in combination form, they can suppress endogenous H_2_S production, which may reduce viability, proliferation, and colony formation, indicating that H_2_S is involved in the growth and progression of human BC.

### 4.2. Inhibition of Endogenous H_2_S Reduces the Migration and Invasion Rate of Human BC Cells

**Figure 2:**
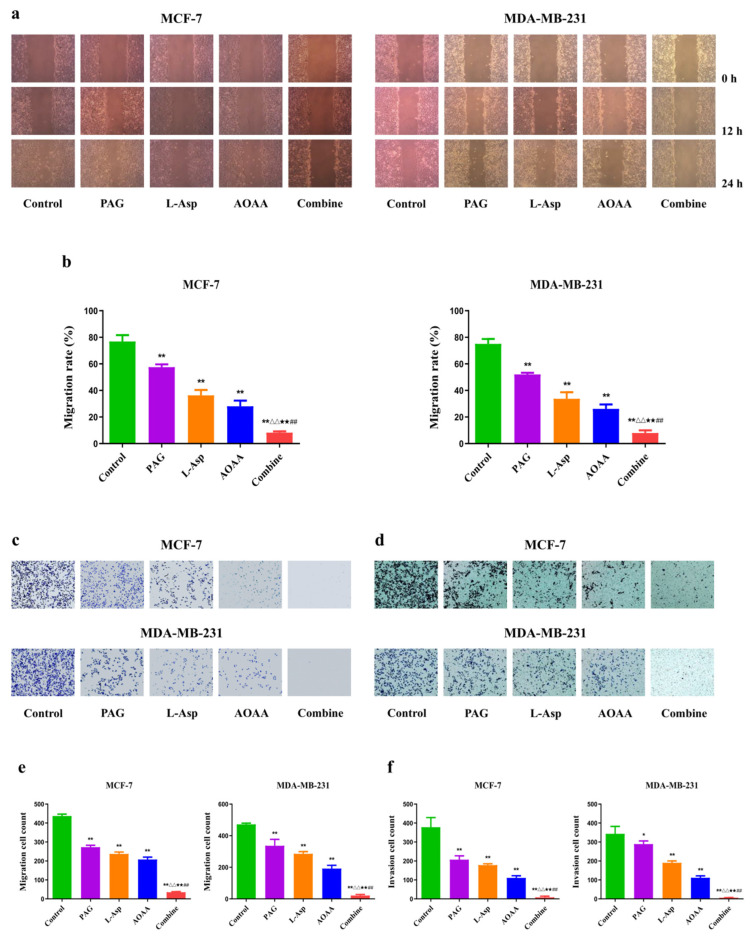
Effects of PAG, AOAA, and L-Asp on the migration and invasion of human BC cells. (**a**) Cell migration was measured by wound-healing assay (original magnification ×100). (**b**) The number of the migrated cells was calculated. (**c**,**d**) Transwell assay was performed to assess the migration and invasion of BC cells (original magnification ×200). (**e**,**f**) The number of the migrated and invasive cells was calculated. The experiments were performed in triplicate. Data are presented as mean ± SEM. ** *p* < 0.01 compared with the control group; ^△△^ *p* < 0.01 compared with PAG group; ^★★^ *p* < 0.01 compared with AOAA group; **^##^** *p* < 0.01 compared with L-Asp group.

Followed by the 24 h treatment, it was observed that the migration and invasion of MCF-7 and MDA-MB-231 cells were reduced in each of the PAG-, L-Asp-, and AOAA-treated groups when compared to the control group. Furthermore, when we used the drugs in combination, a greater inhibitory effect on MCF-7 and MDA-MB-231 cell migration and invasion was recorded compared to the separately treated groups (Figure 2a,b). These results demonstrate that pharmacological treatment with these drugs could significantly inhibit the migration and invasion of human BC cells.

### 4.3. Suppression of Endogenous H_2_S Induces Apoptosis in Human BC Cells

**Figure 3:**
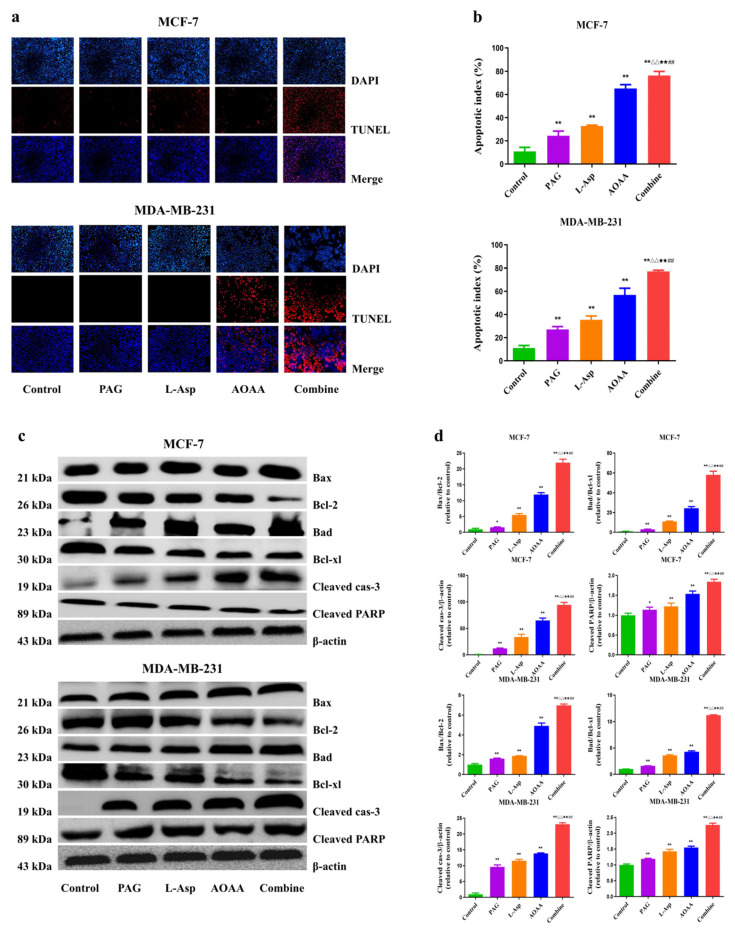
Effects of PAG, AOAA, and L-Asp on the cell death of human BC cells (**a**) The apoptotic level was measured by TUNEL staining (original magnification ×200). (**b**) Apoptotic index was calculated. (**c**) The expression levels of Bax, Bcl-2, Bad, Bcl-xl, cleaved caspase-3, and cleaved PARP were detected by Western blot. β-actin was used as a loading control. (**d**) Densitometric quantification was performed, normalized to the level of β-actin. The experiments were performed in triplicate. Data are presented as mean ± SEM. ** *p* < 0.01 compared with the control group; ^△△^ *p* < 0.01 compared with PAG group; ^★★^ *p* < 0.01 compared with AOAA group; **^##^** *p* < 0.01 compared with L-Asp group.

The results of the present study demonstrate that, compared to the control group, treatment with PAG, L-Asp, or AOAA induce apoptosis in BC cells, as shown in Figure 3a,b. Findings from the TUNEL assay reveal that the combination group has a strong apoptotic index, followed by AOAA, L-Asp, and PAG. Regarding the mitochondrial apoptotic process, which is commonly mediated by cleaved caspase-3 [38], PARP is considered to be a crucial target for exploring this cell death mechanism [39]. In our study, both cleaved caspase-3 and PARP had higher expression in the drug-treated cells compared to the control group. Furthermore, they were higher in the PAG, L-Asp and AOAA separately treated groups than in the control group. Moreover, the amount of these two proteins (cleaved caspase-3 and cleaved PARP) in the combination group was noted to be higher than in the separately treated groups. Collectively, these results clearly indicate the apoptosis induction effect of these drug treatments.

### 4.4. Suppression of Endogenous H_2_S Inhibits Epithelial–Mesenchymal Transition in Human BC Cells

**Figure 4:**
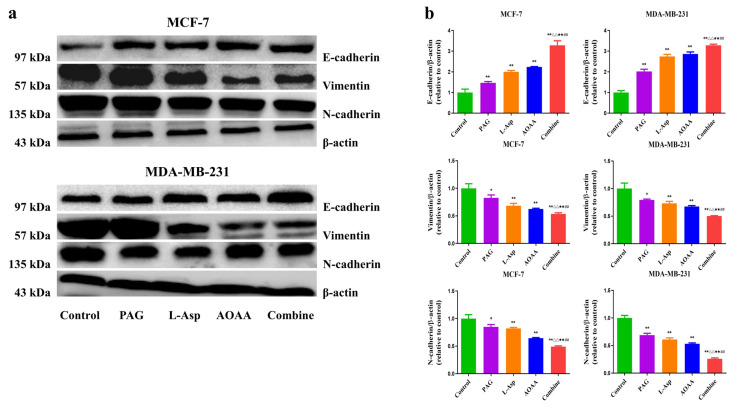
Effects of PAG, AOAA, and L-Asp on the metastasis of ability human BC cells. (**a**) The expression levels of N-Cadherin, N-Cadherin, and Vimentin were detected by Western blot. β-actin was used as a loading control. (**b**) Densitometric quantification was performed, normalized to the level of β-actin. The experiments were performed in triplicate. Data are presented as mean ± SEM. * *p* < 0.05, ** *p* < 0.01 compared with the control group; ^△^ *p* < 0.05, ^△△^ *p* < 0.01 compared with PAG group; ^★^ *p* < 0.05, ^★★^ *p* < 0.01 compared with AOAA group; **^##^** *p* < 0.01 compared with L-Asp group.

Epithelial mesenchymal transition (EMT) has been shown to be crucial in tumorigenesis, where the EMT program enhances metastasis. An aberrant expression of N-cadherin and E-cadherin in many types of tumors is regarded as a hallmark of EMT and considered to be a therapeutic target for inhibiting cancer cell migration [40,41]. Figure 4 shows the EMT rates in BC cells. When PAG, L-Asp, and AOAA were applied for 24 h, there was a significant increase in the expression of E-cadherin and a decrease in N-cadherin and Vimentin compared to the control group. Furthermore, analysis indicates that the effect was much stronger in the combined group, followed by the AOAA, L-Asp, and PAG groups (Figure 4). These findings reveal that suppression of endogenous H_2_S could be a way forward to decrease cancer metastasis in human BC cells. 

### 4.5. Suppression of Endogenous H_2_S Disrupts the PI3K/AKT/mTOR Pathway in Human BC Cells

**Figure 5:**
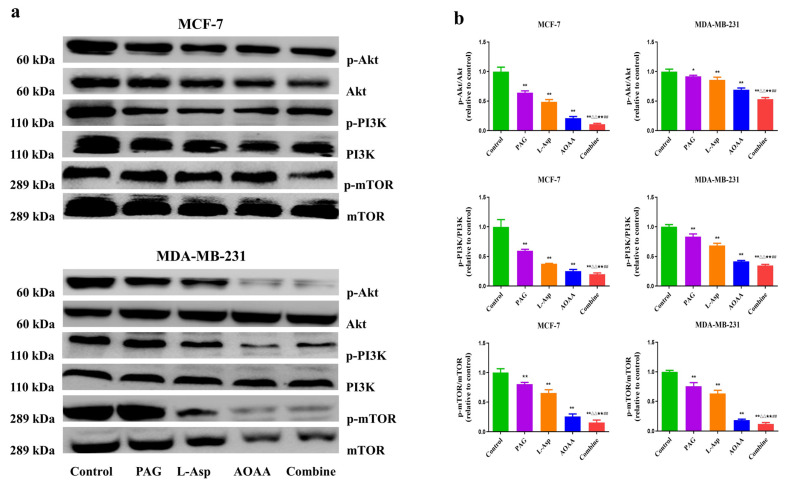
Effects of PAG, AOAA, and L-Asp on the PI3K/AKT/mTOR signaling pathway in human BC cells. (**a**) The expression levels of p-PI3K, p-AKT, and p-mTOR were detected by Western blot. (**b**) Densitometric quantification was performed, normalized to the level of respective non-phosphorylated candidate protein. The experiments were performed in triplicate. Data are presented as mean ± SEM. * *p* < 0.05, ** *p* < 0.01 compared with the control group; ^△^ *p* < 0.05, ^△△^ *p* < 0.01 compared with PAG group; ^★^ *p* < 0.05, ^★★^ *p* < 0.01 compared with AOAA group; **^##^** *p* < 0.01 compared with L-Asp group.

The PI3K/AKT/mTOR signaling pathway is a key signal transduction pathway involved in the many hallmarks of cancer, including survival, metabolism, motility, and genome stability [42,43]. This pathway contributes to several cancer-promoting aspects of the tumor environment, such as angiogenesis and inflammatory cell recruitment [44]. As presented in Figure 5, phosphorylation of PI3K, AKT, and mTOR decreased in the separately treated PAG, L-Asp, and AOAA groups compared to control, which may lead to both decreased cellular proliferation and increased cell death and suppress tumor growth [45]. Overall, these results demonstrate that pharmacological inhibition of endogenous hydrogen sulfide production suppresses BC progression via weakening of AKT, PI3K, and mTOR phosphorylation. 

### 4.6. Suppression of Endogenous H_2_S Inhibits the Angiogenesis and Growth of Human BC Xenograft Tumors

**Figure 6:**
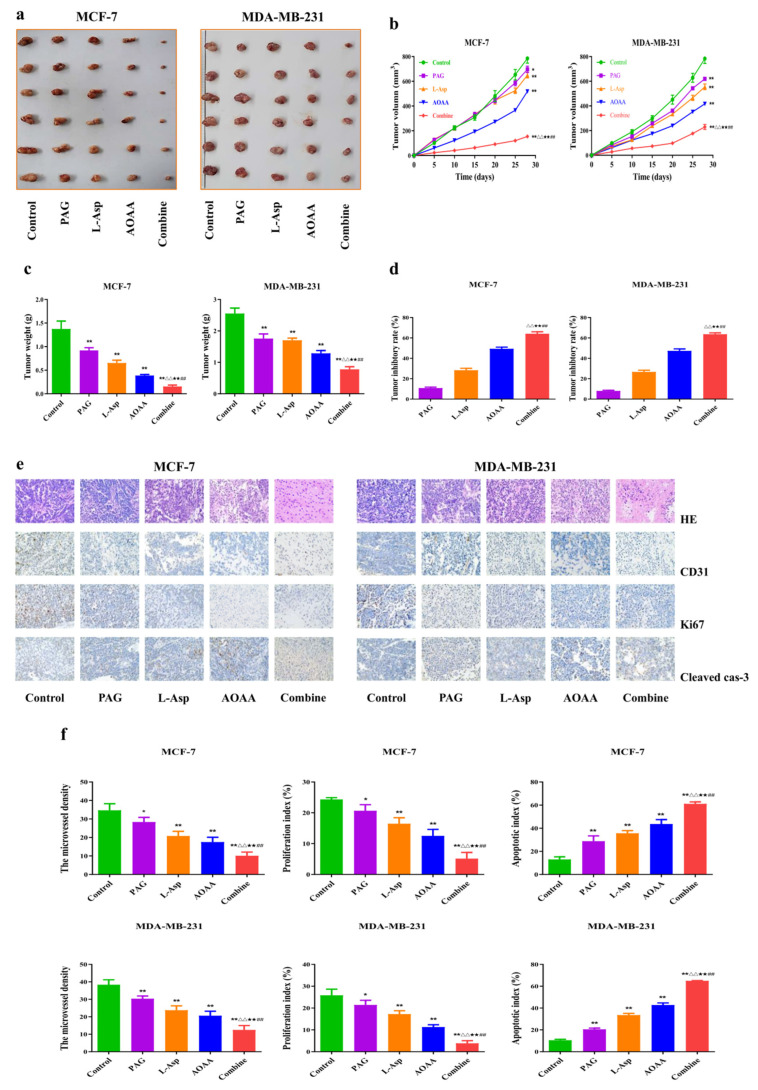
Effects of PAG, AOAA, and L-Asp on the growth of human BC xenograft tumors in nude mice. (**a**) The representative tumor samples from each group are shown. (**b**) The tumor volumes of human BC xenograft tumors were measured (*n* = 6). (**c**) The tumors were weighed (*n* = 6). (**d**) The inhibition rate of tumor growth was calculated (*n* = 6). (**e**) Representative photographs of HE, CD31, Ki67, and cleaved caspase-3 staining in human BC xenograft tumors (original magnification ×400). (**f**) The PI, MVD, and apoptotic index were calculated (*n* = 3). Data are presented as mean ± SEM. * *p* < 0.05, ** *p* < 0.01 compared with the control group; ^△^ *p* < 0.05, ^△△^ *p* < 0.01 compared with PAG group; ^★★^ *p* < 0.01 compared with AOAA group; **^##^** *p* < 0.01 compared with L-Asp group.

Nude mice tumor models have been successfully established using MCF-7 and MDA-MB-231 cells [46,47]. The effect of endogenous H_2_S inhibition on BC xenograft tumor growth was subsequently investigated. The tumor sizes and weights in the PAG, AOAA, and L-Asp groups were significantly lower than in the control group (Figure 6a,b). In addition, the tumor volume and weight were lower in the combination group than in the PAG, AOAA, and L-Asp groups, but the tumor inhibitory rate was higher (Figure 6c,d). It has been observed that HE, CD31, Ki67, and cleaved caspase-3 staining analyses on human BC xenograft tumors also support the effect of drugs, with respect to in vivo results. MVD and PI values were lower in the PAG, AOAA, and L-Asp groups while AI was higher compared to the non-treated control group. Furthermore, in the combination group, the MVD and PI were lower but the AI was higher compared to the PAG, AOAA, and L-Asp groups (Figure 6e,f).

### 4.7. Molecular Docking Analysis

**Figure 7:**
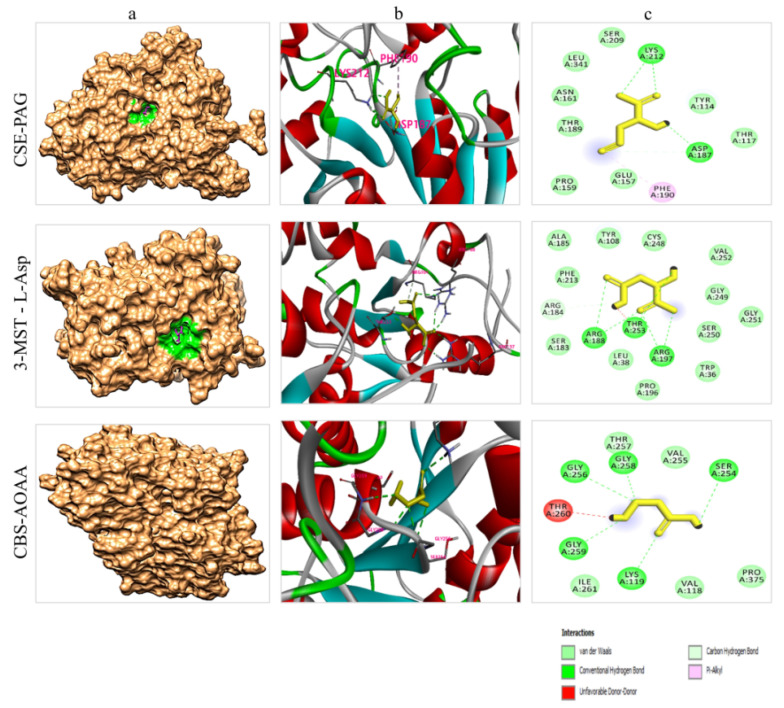
Visualization of drug–protein interaction after molecular docking (**a**) Depiction of each protein docking interaction pocket (magenta: drug molecule, green: drug pocket area in the corresponding enzymes). Note: In the CBS-AOAA complex, the pocket cannot be seen because the drug interaction area is in the cleft of the protein. (**b**) The 3D interaction sites of drugs with corresponding enzymes. (**c**) The 2D interaction of the interacting amino acid residues of the proteins with each drug molecule.

The implementation of computational studies plays a vital role in the early stages of drug discovery and development [48]. In the present study, a molecular docking simulation was carried out to identify the ligand–protein interaction. All the ligands were docked with each of the selected receptor proteins (CSE, CBS, and MPST) to ascertain the optimal conformational binding area by employing the AutoDock Vina tool. This tool provides the minimum binding energy value in kcal/mol, where the lower the binding energy value, the higher the bonding affinity. Also, the greater the number of bonds (i.e., hydrogen and carbon), the stronger the binding interaction between ligand and protein (Table 1). The results of the present study revealed that all the ligands were found in their respective druggable pockets and supported our experimental work findings. Visualization of drug–protein interactions after molecular docking are shown in Figure 7.

## 5. Discussion

H_2_S is known to be a third gaseous molecule that is predominantly involved in various pathophysiological conditions [49,50,51]. Published research has given the utmost importance to the physiological workings of H_2_S in cancer cells. Given the several novel findings on H_2_S donors’ and inhibitors’ roles in cancer [16,52], the rapid development of various H_2_S-based therapeutics reflects the excitement that this unique mediator has sparked, as well as encouraging findings generated in preclinical and early clinical trials. Endogenous H_2_S can promote cancer growth by inducing angiogenesis, regulating mitochondrial bioenergetics, accelerating cell cycle progression, and inhibiting apoptosis. In the present study, we inhibited endogenous H_2_S synthesis by inhibiting H_2_S-generating enzymes in human breast cancer cells.

Breast cancer is one the most frequent malignancies diagnosed in women worldwide [1,2]. There is no promising therapy available that can be used for the medication of individuals presenting advanced stages of breast cancer [53]. Therefore, it is essential to discover putative therapeutic drugs that can be used for the effective management and prevention of breast cancer. In the present study, we investigated the anti-tumor effect of the pharmacological inhibition of H_2_S synthesis by employing the respective inhibitors for H_2_S-generating enzymes in breast cancer in vitro and in vivo, as well as their in silico docking intensities. 

It has been reported that exogenous treatment with PAG and AOAA (1–10 mM) for up to 3 days results in a reduction of cancer cell viability in a dose-dependent manner [52,54]. Adapting to the rationale of published studies, we treated the human BCs (MDA-MB-231 and MCF-7) with 1–10 mM of CSE, MPST, and MPST inhibitors, both individually and in combined solution form. Compared to the untreated cells, our findings demonstrated a remarkable reduction in cancer cell viability and growth rate in treated cells. In the follow-up experiments, we opted for a single concentration (10 mM) and checked the following results. In treated cells, we observed a conspicuous effect on the growth rate, invasion, and migration of BC cells compared to the control group. However, we found that this effect varied in the four groups of treated cells. PAG is the least effective, while the combined group has the strongest and most potent anti-tumor effect against the invasion and migration of both selected types of BC cells. The results showed that pharmacological inhibition of endogenous H_2_S synthesis could reduce the viability, growth, migration, and invasion of both types of human BC cells, empowering the synthesis of H_2_S-based chemopreventive drugs.

Moreover, the molecular docking simulation results also validated the findings of the undertaken study. Results of the binding pockets of each drug molecule predicted by P2RANK web servers were similar to those previously reported by studies on these enzymes. In a previous study carried out by Sun et al. (2009), in addition to its structural basis, they reported the inhibition mechanism of the CSE enzyme [55]. They documented that CSE is a tetramer when it is in PLP (pyridoxal-5-phosphate)-bound states. Each monomer of CSE contains two domains: (a) a larger PLP-binding domain ranging from 9 to 263 residues, and (b) a smaller domain covering 264–401 residues. The PAG bound to the CSE enzyme occupies the space of the substrate’s side chain and covalently binds to the Tyr114 residue, which is thought to block substrate accessibility, thereby inhibiting CSE activity. Additionally, during this inhibition, Asp187 and Lys212 facilitate this process to accomplish the task [55]. Likewise, Meier et al. (2001) conducted a study to elucidate the structure of the CBS enzyme and its mechanism of inhibition [56]. They reported the crucial roles of Lys119, Gly256, Thr258, Gly259, and Thr260 residues in the active site of the enzyme. They revealed that these amino acid residues have significant importance in the working or inhibition process of the CBS enzyme [56]. Similarly, Yadav et al. (2013) extensively studied the structure of the MPST enzyme and highlighted the roles of Tyr108, Arg188, Arg197, and Thy253 amino acid residues in the functional behavior of this enzyme [57]. Overall, our drug–protein docking analysis explained the selectively and competitiveness of these drugs in inhibiting enzyme physiology, enhancing the reliability of these drugs to be used as future anti-cancer medicines. It was observed that PAG had the highest binding affinity (−5.4 kcal/mol) towards the CSE protein, followed by L-Asp and AOAA. Similarly, L-Asp was found to have the best binding interaction, with a binding energy of −5.3 kcal/mol, compared to other inhibitors with MPST protein. Likewise, AOAA was found to be good inhibitor of CBS protein and had a binding energy of −4.6 kcal/mol (Table 1). Figure 7 depicts the types of bonds and interacting amino acid residues in the ligand–protein interactions. The overall results of all the selected inhibitors’ binding affinities with the three selected proteins are moderately convincing and support our experimental work findings.

Furthermore, apoptosis plays a crucial role in the progression and dynamic balance of complex organization in multicellular organizations [58]. At the cellular level, apoptosis occurs via both intrinsic and extrinsic pathways, wherein the former is mediated by mitochondria and the latter is stimulated by death receptors. This results in numerous morphological modifications, including genetic material condensation, nuclear fragmentation, and cell shrinkage [59]. Proapoptotic proteins (such as Bad and Bax) and members of the Bcl-2 family (such as Bcl-xl and Bcl-2) are the primary mediators of intrinsic cell death. However, caspases and PARP activation by certain stimuli also contribute to the occurrence of apoptotic cascades in the cell [39,58]. Going through the published literature, it has been shown that AOAA and PAG treatment causes cell death by promoting apoptotic cascades in the A549 and 95D human non-small cell lung cancer cells [13,60].

In the present study, following treatment, we observed the ratios of Bad/Bax-xl and Bax/Bcl-2 and the increased expression at the protein level of cleaved PARP and caspase-3 in the PAG, L-Asp, and AOAA groups. The combined treatment cells exhibited a stronger effect on the ratios of Bad/Bax-xl and Bax/Bcl-2 and higher protein expression of cleaved PARP and caspase-3 than the PAG, L-Asp, and AOAA groups. These results suggest that inhibition of endogenous H_2_S synthesis induces apoptosis in human BC cells.

Similarly, following the 24 h treatment of PAG, L-Asp, and AOAA, significant downregulation of N-cadherin and Vimentin and increased expression of E-cadherin were observed compared to the control group. We found a more potent effect in the combined group, followed by the AOAA-treated group. Considering the inevitable roles of E and N cadherins in cancer metastasis [40,41], these findings reveal that suppression of endogenous H_2_S could be a way forward for decreasing cancer metastasis in human BC cells.

The PI3K/AKT/mTOR pathway is essential in the regulation of cell growth, viability, mortality, and protein synthesis [61]. Research reveals that activation of this pathway contributes to tumor predisposition and increased risk of death [62]. It has also been found that inhibition of PI3K/AKT/mTOR leads to a decrease in proliferation index and shows potent efficacy in the treatment of BC [63]. Our findings show that PAG, L-Asp, and AOAA exposure disrupt PI3K/AKT/mTOR and reduce its level compared to the control group. Following the apoptosis result trends, we observed that the combined group has a potent inhibitory effect on the downregulation of the PI3K/AKT/mTOR pathway. Taken together, these results show that inhibition of endogenous H_2_S decreases the expression of p-PI3K, p-mTOR, and p-AKT, indicating that they inhibit the proliferation, invasion, and migration of human BC cancer cells via blocking the PI3K/AKT/mTOR pathway. In recent research studies, MDA-MB-231 and MCF-7 cells have been successfully used to establish subcutaneous xenograft models [46,47]. Previously, the effect of endogenous H_2_S inhibition by employing AOAA and PAG on xenograft tumor growth has been reported in human chronic myeloid leukemia and astrocytoma. They observed dramatically decreased tumor growth in the treated animals compared to control [64,65]. Our study showed that PAG, AOAA, and L-Asp significantly inhibited BC xenograft tumor growth, and the combined group showed stronger preventive effects on tumor growth.

Studies have established the fact that CD31 is a key biomarker for vascular endothelial cells, and that tumor MVD may be reflected in CD31 stains [30]. Similarly, Ki67 and cleaved caspase-3 are well known for detecting proliferation index and the apoptotic index, respectively [31,32]. Supporting the trends, our IHC results of tumor tissue show that PAG, L-Asp, and AOAA reduced CD31 and Ki67 levels but increased cleavage caspase-3 levels. In addition, the combined group showed lower expression of CD31 and elevated levels of cleavaged-caspase-3 than PAG, L-Asp, and AOAA groups.

## 6. Conclusions

In conclusion, the results of the present study suggest that reducing endogenous H_2_S levels by pharmacologically inhibiting H_2_S-producing enzymes with their respective synthesized inhibitors attenuates the growth of human BC cells in vitro and in vivo. H_2_S inhibition leads to enhanced cell death and disrupts the PI3K/AKT/mTOR pathway, which eventually reduces the progression of BC cells. The docking potentials of these drugs to the active sites of their respective enzymes are consistent with the wet lab experiments, enhancing the reliability of these drugs. These findings, especially those for the combined dose with treatments of PAG, L-Asp, and AOAA, give a new insight towards suppressing H_2_S levels in cells to control the progression of tumors. Given the pervasive nature of H_2_S and the rapid development of various H_2_S-based therapeutics approaches, the present study supports the findings from preclinical and early clinical trials and strongly endorses the rationale of empowering H_2_S-based chemo-preventive drug synthesis against different malignancies. It is worth exploring the development of innovative H_2_S-based therapeutic and diagnostic approaches for pre-clinical findings, which hold the promise of increased efficacy, lower toxicity, or both, to start trials in clinical settings.

## Figures and Tables

**Table 1:** The binding energy value (in kcal/mol) of drugs (PAG, L-Asp, and AOAA) to their respective enzymes (CSE, 3-MPST, and AOAA) in the drug docking exercise.

Drug	PubChem ID	Cystathionine Gamma-Lyase	3-Mercaptopyruvate Sulfurtransferase	Cystathionine Beta-Synthase
L-aspartic acid	5960	−4.9	−5.3	−4.5
DL-Propargylglycine	95575	−5.4	−5.1	−4.8
Aminooxyacetic acid	286	−4	−4.6	−4.6

## Data Availability

All data generated or analyzed in this study were included in this article.

## Supplementary Materials

The following supporting information can be downloaded at: https://www.mdpi.com/article/10.3390/molecules27134049/s1, Figure S1: Dose dependent effects of PAG, AOAA, and L-Asp on viability of human breast cancer cells (MCF-7, MDA-MB-231); Figure S2: An intra- dose effect comparison of cell viability between combined low concentration (10 mL/mol) of PAG, AOAA, and L-Asp and higher concentration of individual drugs (30 Mm) by using a CCK-8 test on human breast cancer cells (MCF-7, MDA-MB-231); Table S1.

## Abbreviations

H_2_S—Hydrogen sulfide; L-Cys—L-cysteine; CSE—Cystathionine-γ-lyase; CBS—Cystathionine-β-synthase; 3-MST—3-Mercaptopyruvate sulfurtransferase; CAT—Cysteine aminotransferase; BC—Breast cancer; PAG—DL-propargylglycine; AOAA—Aminooxyacetic acid; L-Asp—L-aspartic acid; PBS—Phosphate buffer saline; MTT—3-(4,5)-Dimethylthiahiazo(-z-y1)-3,5-di-phenytetrazoliumromide; EdU—5-Ethynyl-2′-deoxyuridine; MR—Migration rate; TUNEL—TdT-mediated dUTP-biotin nick end labeling; Bcl-xl—B-cell lymphoma-extra-large; PI3K—hosphatidylinositol-3-Kinase; Bcl-2—B-cell lymphoma-2; Bad—Bcl-xl/Bcl-2-associated death promoter; Bax—Bcl-2-associated X protein; PARP—Poly adenosine diphosphate-ribose polymerase; CST—Cell Signaling Technology; mTOR—mammalian target of rapamycin; IR—Inhibition rate; HE—Hematoxylin and eosin; IHC—Immunohistochemistry; CD31—Cluster of differentiation 31; SEM—Standard error of the mean.
