# Supplementary material for: Pharmacological Inhibition of Endogenous Hydrogen Sulfide Attenuates Breast Cancer Progression
Source: Molecules. 2022 Jun 23;27(13):4049. doi: 10.3390/molecules27134049 (PMC9268373; doi:10.3390/molecules27134049)
Supplement: Supplementary file 1 [file molecules-27-04049-s001.zip › Supplementary Materials.pdf]

Pharmacological Inhibition of Endogenous Hydrogen Sulfide Attenuates Breast Cancer Progression

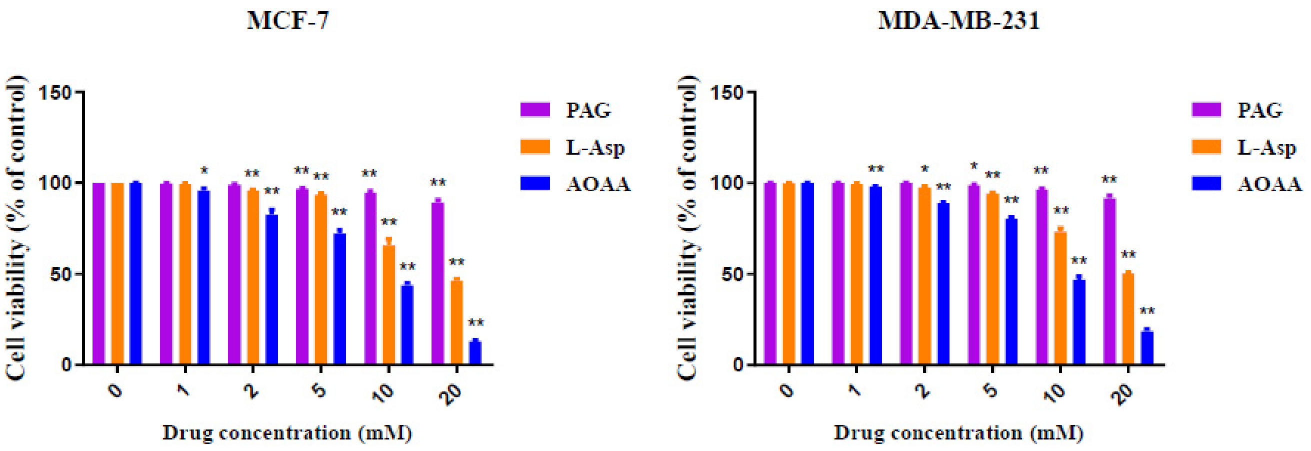

Figure S1. Dose dependent effects of PAG, AOAA, and L-Asp on viability of human breast cancer cells (MCF-7, MDA-MB-231).

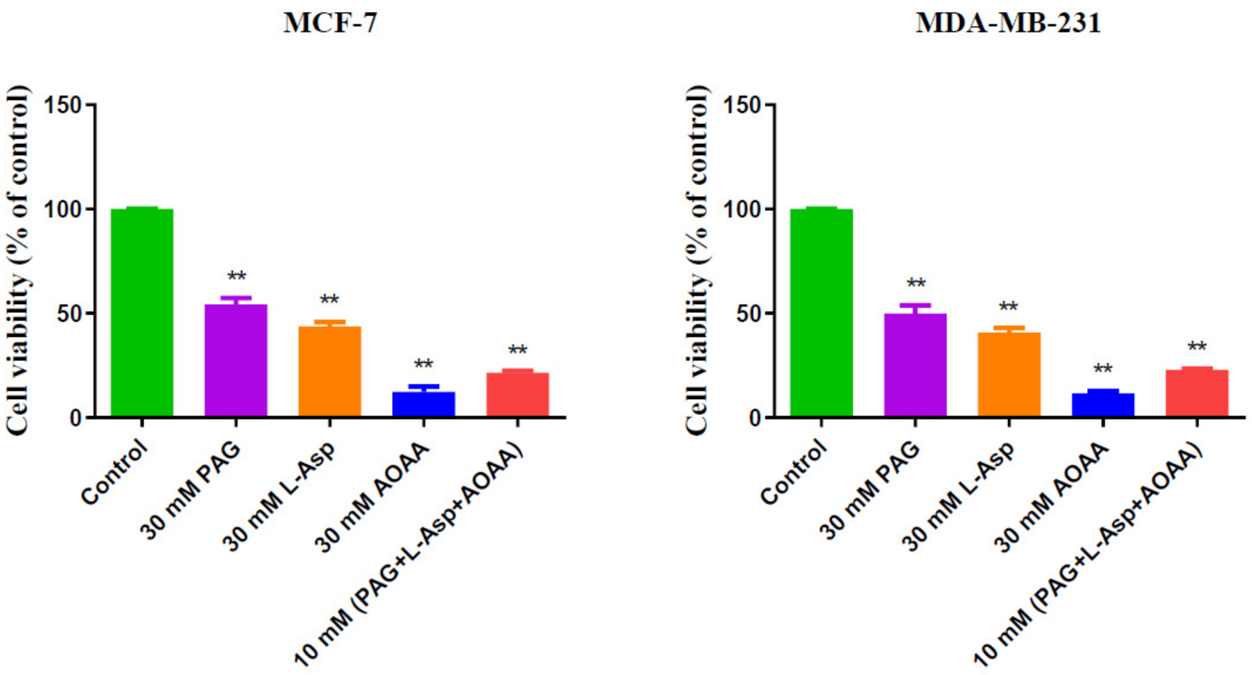

Figure S2. An intra- dose effect comparison of cell viability between combined low concentration (10ml/mol) of PAG, AOAA, and L-Asp and higher concentration of individual drugs (30 Mm) by using a CCK-8 test on human breast cancer cells (MCF-7, MDA-MB-231).
